# The Law of Abortion

**Published:** 1875-09

**Authors:** 


The Law of Abortion.—A week or two since an irregular physician of this city was convicted of being an "accessory after the act" in a case of abortion followed by death. He delivered the body to an irregular college of this city, which led to the detection of the crime. Judge Finletter, in summing up the case to the jury, made the following remarks on the relation of medical men to the law:

"The law invests the physician with peculiar privileges. In the exercise of his profession, all his acts must be considered lawful until the contrary be shown. If he be administering anywhere, the presumption is that he is lawfully there for a lawful purpose, to which he was regularly called. If it be shown that a miscarriage was produced, it must be presumed that he had nothing to do with it until it be shown that he participated in it, or counseled, or advised, or directed it. Even if it be shown that he produced it, the presumption would be that it was lawfully done, until the contrary be established. In this connection, however, it must not be forgotten that an intentional miscarriage, for the purpose only of producing a miscarriage, is always unlawful. These presumptions of innocence are intended to protect the honest members of a profession whose glory it is to answer the call of suffering with a promptitude which forbids an inquiry into the surroundings of a patient, or guarding themselves with witnesses in the performance of their duties. They are not, however, to be means of covering up crime and protecting criminals. Nor should they be extended to acts of the physician not within the scope of his professional services. With the life of his patient his responsibility ends, and his subsequent acts and conduct are to be considered and adjudged as those of other men. It would be a rare exigency, indeed, which could justify him in appropriating for dissection the body of his patient. How far the profession should be on the alert to suppress the crime of abortion, to expose and bring to punishment the perpetrators, to aid with all their great intelligence and opportunities of observation the ministers of the law, must be left for them to determine. Although it may not be necessary, I regard it as a part of my duty to call your attention to the character of the crime charged against these defendants. There is none which equals it in wickedness, or in its terrible consequences. It is greatly to be feared that abortion is no longer confined to the victims of passion and seduction. It stalks abroad so brazenly heralded, that childhood in its most guarded home may see and feel its corrupting influences. That it breaks down the guards of public and private chastity, and fills the ranks of prostitution, is the smallest of its evils. When it enters the domestic circle, all that makes home holy and blessed withers in its track; purity of thought and act, the respect and confidence of man and wife, the pride and glory of children, and their tender care, and all regard for family duties and responsibilities, disappear forever. *Medical and Surgical Reporter.*

